# Phenotype-specific differences in mortality and cerebrovascular risk in narcolepsy type 1 and type 2: a real-world propensity score-matched cohort study

**DOI:** 10.1097/MS9.0000000000005502

**Published:** 2026-10-06

**Authors:** Naveen Gautam, Anuja Upadhyay, Bal Krishna Subedi, Michael S. Weinstein, Gina Stefanelli, Michael Cerino, Daniel Bitetto

**Affiliations:** a Department of General Medicine, Gulmi Durbar Basic Hospital, Gulmi, Nepal; b Department of Internal Medicine, Jefferson Einstein Montgomery Hospital, East Norriton, Pennsylvania, USA; c Pulmonary, Critical Care, and Sleep Medicine, Advocare Pulmonology and Sleep Medicine, Lansdale, Pennsylvania, USA

**Keywords:** cohort study, ischemic stroke, mortality, narcolepsy type 1, narcolepsy type 2, United States

## Abstract

**Background:**

Narcolepsy type 1 (NT1) and narcolepsy type 2 (NT2) are clinically distinct disorders, but comparative long-term data on hard outcomes remain limited. We compared all-cause mortality and major cerebrovascular and cardiovascular outcomes between NT1 and NT2 in a large real-world cohort.

**Materials and methods:**

This retrospective cohort study used the TriNetX US Collaborative Network, with analysis performed on 28 September 2025. Adults aged 18–75 years with NT1 (ICD-10-CM G47.411) or NT2 (ICD-10-CM G47.419) were identified. After 1:1 propensity score matching for demographics, comorbidities, lifestyle-related factors, and medication exposures, 19 907 matched pairs were analyzed. The primary outcome was all-cause mortality over 10 years. Secondary outcomes were ischemic stroke, major adverse cardiovascular events (MACE), arrhythmia, and sudden cardiac death. Hazard ratios (HRs) with 95% confidence intervals (CIs) were derived within the TriNetX analytics platform.

**Results:**

NT1 was associated with lower all-cause mortality than NT2 (1.57% vs 2.01%; HR 0.764, 95% CI: 0.659–0.885; *P* < 0.001). NT1 was also associated with a higher risk of ischemic stroke (1.49% vs 1.23%; HR 1.193, 95% CI: 1.006–1.415; *P* = 0.042). There were no statistically significant differences in MACE (HR 1.084, 95% CI: 0.941–1.249; *P* = 0.264), arrhythmia (HR 0.864, 95% CI: 0.740–1.009; *P* = 0.065), or sudden cardiac death (HR 0.844, 95% CI: 0.522–1.365; *P* = 0.489).

**Conclusion:**

In this matched real-world cohort, NT1 was associated with lower all-cause mortality but higher ischemic stroke risk compared with NT2. Because this was an observational analysis, these findings represent associations rather than causal effects and should be interpreted as hypothesis-generating; they support phenotype-aware risk assessment, especially the prospective validation of cerebrovascular risk in NT1.

## Introduction

Narcolepsy is a chronic neurologic sleep–wake disorder characterized by excessive daytime sleepiness and dysregulation of rapid eye movement sleep. Clinically, it is divided into narcolepsy type 1 (NT1), usually associated with cataplexy and hypocretin-1 deficiency, and narcolepsy type 2 (NT2), a phenotype with less well-defined and likely heterogeneous biology^[^1,2^]^. Narcolepsy is an uncommon disorder, affecting on the order of 25 to 50 persons per 100 000 in general-population estimates^[^3^]^. NT1, defined by cataplexy and hypocretin-1 deficiency, has historically been more precisely characterized than NT2, whereas reported estimates for NT2 are more variable, in part because of its less specific diagnostic criteria and its recognized tendency to be reclassified over time^[^3,4^]^. Because the two phenotypes differ in their underlying neurobiology, accurate phenotype differentiation is increasingly emphasized for prognosis, comorbidity screening, and treatment selection; however, the downstream consequences of this distinction for hard cardiovascular and cerebrovascular outcomes remain incompletely defined.

HIGHLIGHTS

- A large real-world propensity score-matched cohort study compared narcolepsy type 1 and type 2 over 10 years.
- Narcolepsy type 1 was associated with a lower all-cause mortality than narcolepsy type 2.
- Narcolepsy type 1 was associated with a higher risk of ischemic stroke.
- No significant differences between phenotypes were observed for major adverse cardiovascular event, arrhythmia, or sudden cardiac death.
- These findings are hypothesis-generating and support phenotype-aware vascular risk assessment.

The hypocretin–orexin system influences not only wakefulness but also autonomic tone, metabolic regulation, and cardiovascular homeostasis^[^5,6^]^. Accordingly, prior studies have linked narcolepsy, particularly NT1, with obesity, dyslipidemia, and other metabolic comorbidities^[^7,8^]^. Even so, whether these differences translate into divergent risks of stroke, major cardiovascular events, or death between phenotypes remains uncertain.

Population-based and registry studies suggest that narcolepsy as a whole is associated with excess mortality and elevated cardiovascular burden^[^9–11^]^. However, pooled analyses may obscure phenotype-specific differences, especially given the diagnostic instability of NT2 and heterogeneity of patients categorized within that phenotype^[^4,12^]^. Clarifying phenotype-specific risk has potential relevance for risk stratification, preventive care, and the use of large-scale health informatics in sleep medicine^[^5,13,14^]^.

Using the TriNetX global federated research network, we conducted a large real-world, propensity score-matched comparison of NT1 and NT2 to evaluate all-cause mortality, ischemic stroke, and major cardiovascular outcomes over 10 years^[^15^]^. We hypothesized that the two phenotypes would demonstrate different long-term outcome patterns. This cohort study has been reported in line with the STROCSS 2025 guideline^[^16^]^.

## Materials and methods

### Study design, data source, and ethical approval

This was a retrospective observational cohort study using the TriNetX US Collaborative Network, a federated electronic health record (EHR) research platform that aggregates de-identified data from participating healthcare organizations across the United States^[^15^]^. The analysis was performed on 28 September 2025. Because TriNetX provides only de-identified aggregate data and no directly identifiable patient information, formal Institutional Review Board approval was not required for this study, and individual informed consent was not required.

### Participants and cohort definition

We identified adults aged 18–75 years with a diagnosis of NT1, defined by ICD-10-CM code G47.411 (narcolepsy with cataplexy), or NT2, defined by ICD-10-CM code G47.419 (narcolepsy without cataplexy). The index date was the first recorded diagnosis of the phenotype of interest. To improve the temporal relevance of the cohort, patients whose index date occurred more than 20 years before the analysis date were excluded.

The initial query identified 20 131 patients with NT1 and 59 611 patients with NT2. After 1:1 propensity score matching, 19 907 patients remained in each cohort for the primary matched analysis (Fig. 1).

**Figure 1.**
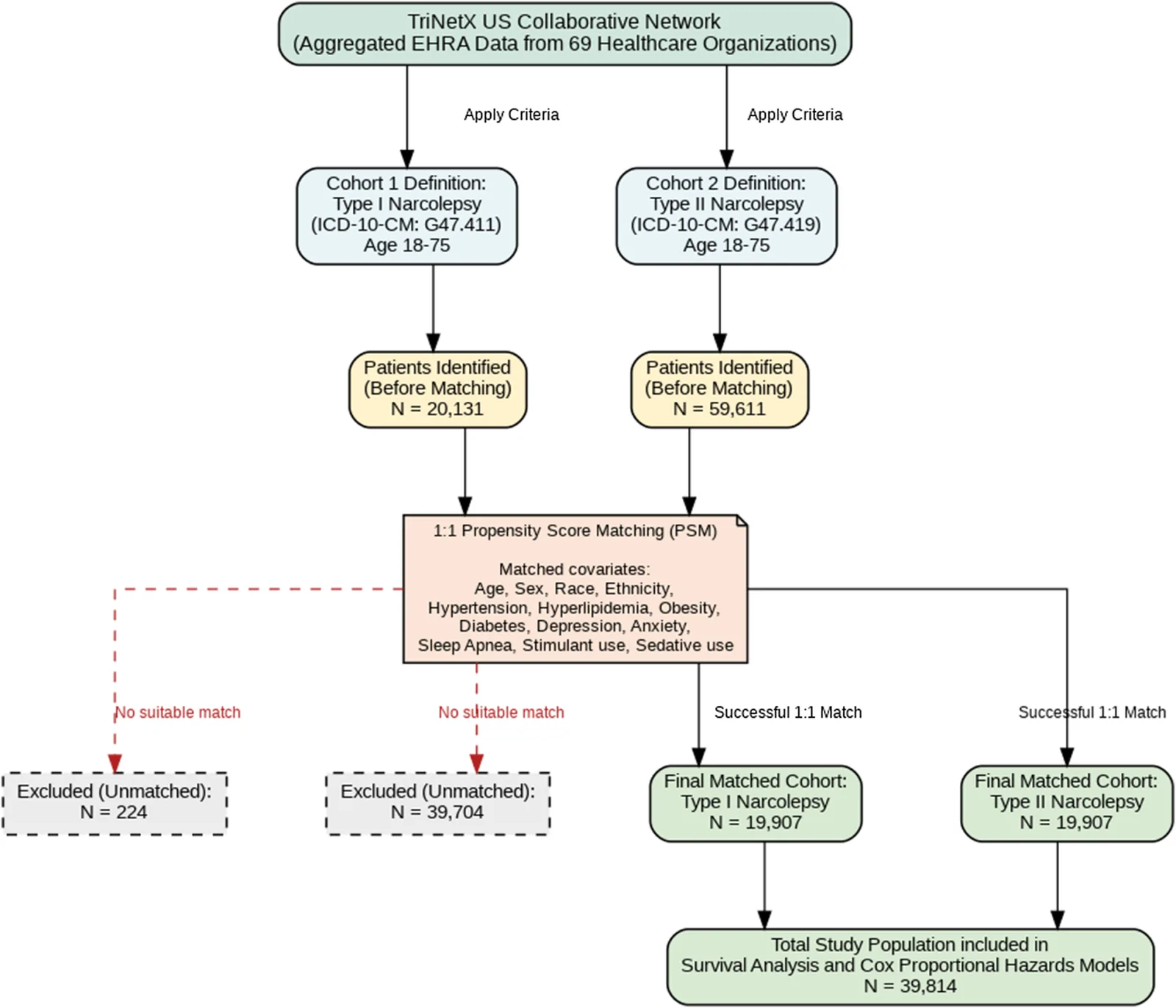
Flow diagram of cohort identification and propensity score matching. Adults aged 18–75 years with narcolepsy type 1 or narcolepsy type 2 were identified in the TriNetX US Collaborative Network, and this was followed by 1:1 propensity score matching to generate the final matched cohort of 19 907 patients per group.

### Outcomes

The primary outcome was all-cause mortality, defined by a deceased status in the demographic record. Secondary outcomes were ischemic stroke (ICD-10-CM code I63), major adverse cardiovascular events (MACE, a composite of cerebral infarction, non-ST-elevation myocardial infarction, or ST-elevation myocardial infarction), arrhythmia (atrial fibrillation, ventricular tachycardia, or ventricular fibrillation), and sudden cardiac death, identified by a personal history of sudden cardiac arrest (Z86.74).

Outcomes were assessed from 1 day after the index date up to 3650 days (10 years). For each specific outcome analysis, patients with that outcome documented before the start of follow-up were excluded from the corresponding risk set to favor the assessment of incident events.

As exploratory negative control outcomes, we also evaluated dementia, depression, anxiety, hypothyroidism, and type 1 diabetes mellitus to assess whether observed differences might simply reflect broad surveillance effects rather than phenotype-specific risk.

### Covariates and statistical analysis

To reduce confounding, 1:1 propensity score matching was performed within the TriNetX analytics platform. Propensity scores were estimated using a logistic regression model in which narcolepsy phenotype (NT1 versus NT2) was the dependent variable and the prespecified covariates were the predictors. Matching used a greedy nearest-neighbor algorithm with a caliper of 0.1 of the pooled standard deviation of the logit of the propensity score and was performed in a 1:1 ratio without replacement; the platform randomized the order of records to avoid order-dependent bias inherent to nearest-neighbor matching. Because clinical covariates were derived from coded electronic health record data, the absence of a diagnosis, procedure, or medication code was treated as the absence of that characteristic rather than as missing data. The matching covariates comprised demographic factors (age and sex), race and ethnicity, cardiometabolic and psychiatric comorbidities (hypertension, hyperlipidemia, obesity, diabetes mellitus, sleep apnea, depression, and anxiety), and medication exposures, including wake-promoting agents and stimulants (e.g., modafinil and amphetamines), sodium oxybate, sedatives (e.g., benzodiazepines), and antihypertensive and other cardiovascular medications. Sex was explicitly included among the matching variables. Covariate balance after matching was assessed using standardized mean differences (SMDs), with SMD values below 0.1 considered acceptable balance. The full set of matching covariates with SMDs before and after matching is provided in Supplemental Digital Content Table S1, available at: https://links.lww.com/MS9/B413, and balance is also displayed graphically in Figure 2.

**Figure 2.**
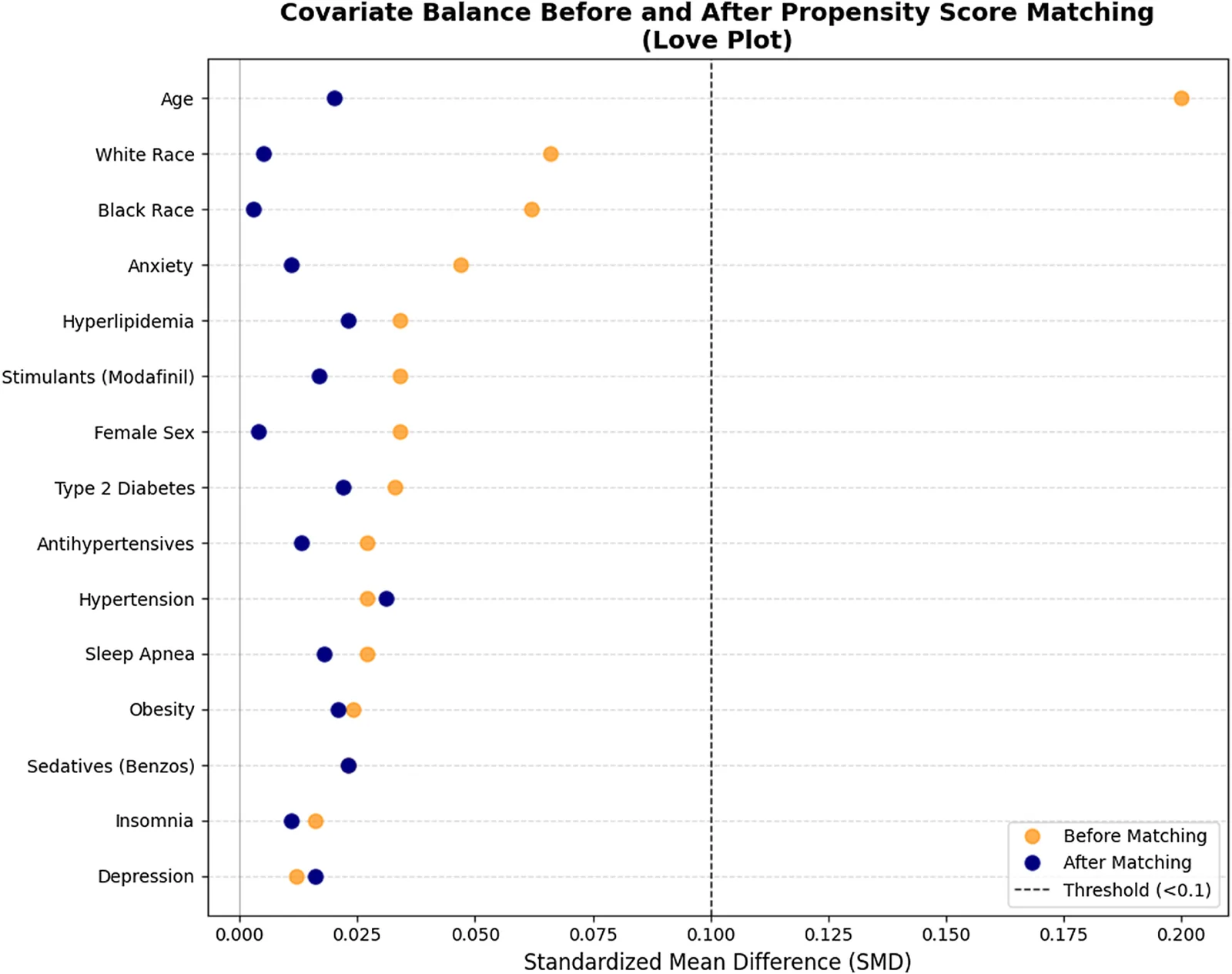
Love plot of covariate balance before and after propensity score matching. The dashed vertical line indicates an SMD threshold of 0.1. All matched covariates were below this threshold after matching, indicating adequate balance between the cohorts.

Time-to-event outcomes were evaluated with Kaplan–Meier methods and compared using log-rank testing. Hazard ratios (HRs) with 95% confidence intervals (CIs) were estimated on the TriNetX platform. Statistical significance was defined as a two-sided *P*-value below 0.05. Given the retrospective design, reliance on coded EHR data, and potential for residual confounding, results were interpreted as associations rather than causal effects.

## Results

### Study population and matching

After matching, the study population comprised 39 814 adults, including 19 907 patients with NT1 and 19 907 with NT2. Baseline characteristics were well balanced after matching, with all matched covariates demonstrating SMD values below 0.1. The matched mean age was 42.8 ± 15.1 years in the NT1 cohort and 42.5 ± 14.8 years in the NT2 cohort. Women accounted for 65.8% of the NT1 cohort and 66.0% of the NT2 cohort. Detailed baseline characteristics are presented in Table 1, and the matching flow is summarized in Figure 1.

**Table 1:** Baseline characteristics of the matched narcolepsy type 1 and narcolepsy type 2 cohorts.

Characteristic	Narcolepsy type 1 (*n* = 19 907)	Narcolepsy type 2 (*n* = 19 907)	*P* value	Standardized mean difference
**Demographics**				
Age, mean ± SD, years	42.8 ± 15.1	42.5 ± 14.8	0.044	0.020
Female sex, no. (%)	13,106 (65.8%)	13,148 (66.0%)	0.657	0.004
White race, no. (%)	13,480 (67.7%)	13,438 (67.5%)	0.653	0.005
Black or African American race, no. (%)	2957 (14.9%)	2936 (14.7%)	0.767	0.003
**Comorbidities**				
Hypertension, no. (%)	3123 (15.7%)	2900 (14.6%)	0.002	0.031
Hyperlipidemia, no. (%)	1903 (9.6%)	1768 (8.9%)	0.019	0.023
Obesity, no. (%)	3192 (16.0%)	3040 (15.3%)	0.036	0.021
Diabetes mellitus, no. (%)	1443 (7.2%)	1332 (6.7%)	0.029	0.022
Sleep apnea, no. (%)	4439 (22.3%)	4287 (21.5%)	0.066	0.018
Depression, no. (%)	3940 (19.8%)	3811 (19.1%)	0.103	0.016
Anxiety, no. (%)	3755 (18.9%)	3671 (18.4%)	0.280	0.011
**Medication exposure**				
Modafinil, no. (%)	2302 (11.6%)	2194 (11.0%)	0.087	0.017
Sodium oxybate, no. (%)	793 (4.0%)	745 (3.7%)	0.212	0.013
Amphetamine stimulants, no. (%)	2429 (12.2%)	2304 (11.6%)	0.053	0.019

SD, standard deviation. An SMD below 0.1 indicates acceptable covariate balance after matching.

### Primary outcome

Over 10 years of follow-up, NT1 was associated with a lower risk of all-cause mortality than NT2. There were 312 deaths among 19 893 at-risk patients in the NT1 cohort (1.57%) compared with 400 deaths among 19 892 at-risk patients in the NT2 cohort (2.01%), corresponding to an HR of 0.764 (95% CI: 0.659–0.885; *P* < 0.001). Kaplan–Meier analysis demonstrated separation of the survival curves favoring NT1 (Fig. 4).

### Secondary cardiovascular and cerebrovascular outcomes

NT1 was associated with a higher risk of ischemic stroke than NT2. There were 293 stroke events among 19 620 at-risk patients in the NT1 cohort (1.49%) and 241 events among 19 610 at-risk patients in the NT2 cohort (1.23%), corresponding to an HR of 1.193 (95% CI: 1.006–1.415; *P* = 0.042).

No statistically significant between-group differences were observed for MACE, arrhythmia, or sudden cardiac death. For MACE, the HR was 1.084 (95% CI: 0.941–1.249; *P* = 0.264). For arrhythmia, the HR was 0.864 (95% CI: 0.740–1.009; *P* = 0.065). For sudden cardiac death, the HR was 0.844 (95% CI: 0.522–1.365; *P* = 0.489). A summary of these associations is provided in Table 2 and Figure 3.

**Figure 3.**
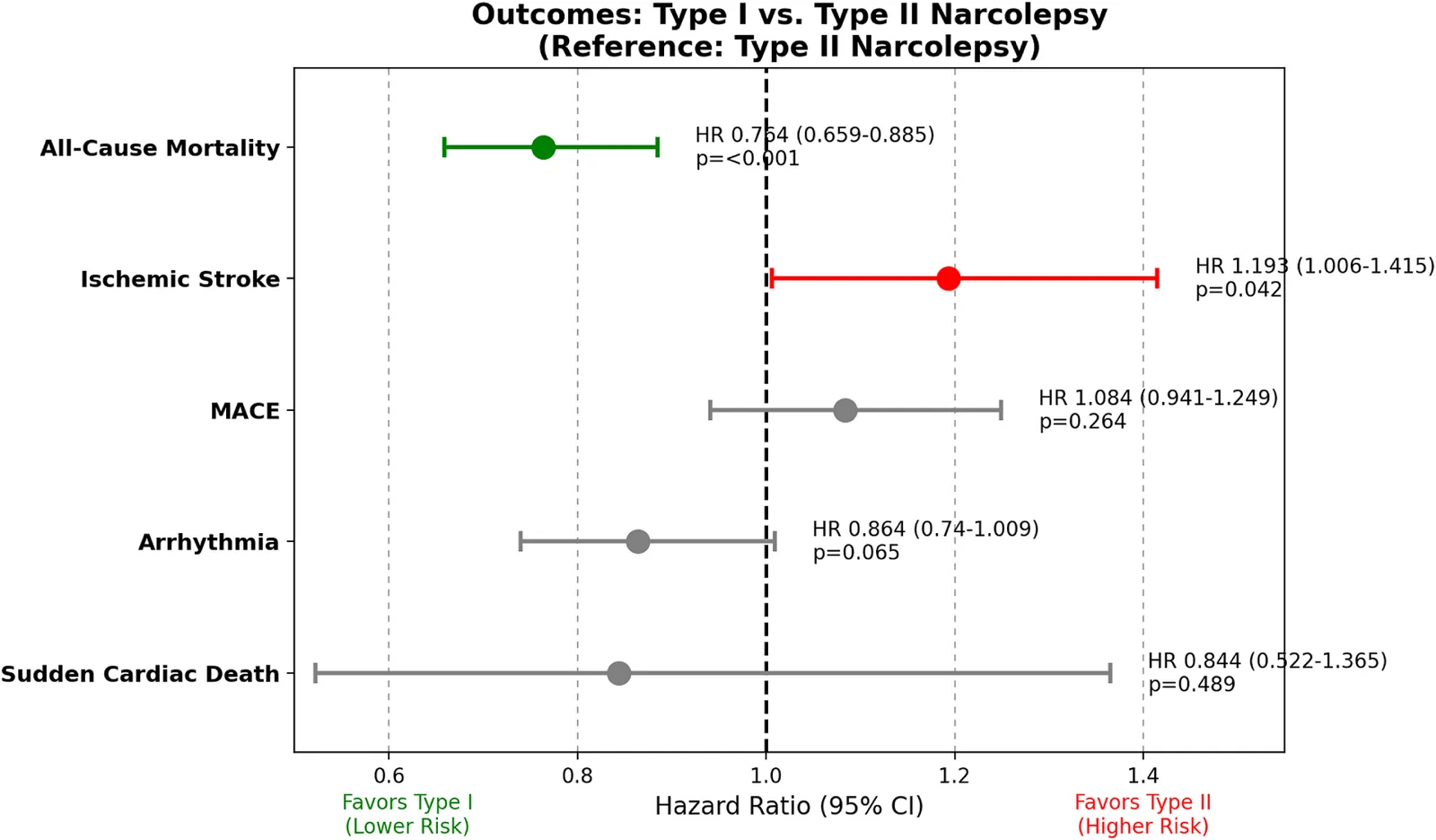
Forest plot of hazard ratios for primary and secondary outcomes comparing narcolepsy type 1 with narcolepsy type 2. Values to the left of 1.0 indicate a lower risk for narcolepsy type 1; values to the right indicate a higher relative risk for narcolepsy type 1.

**Figure 4.**
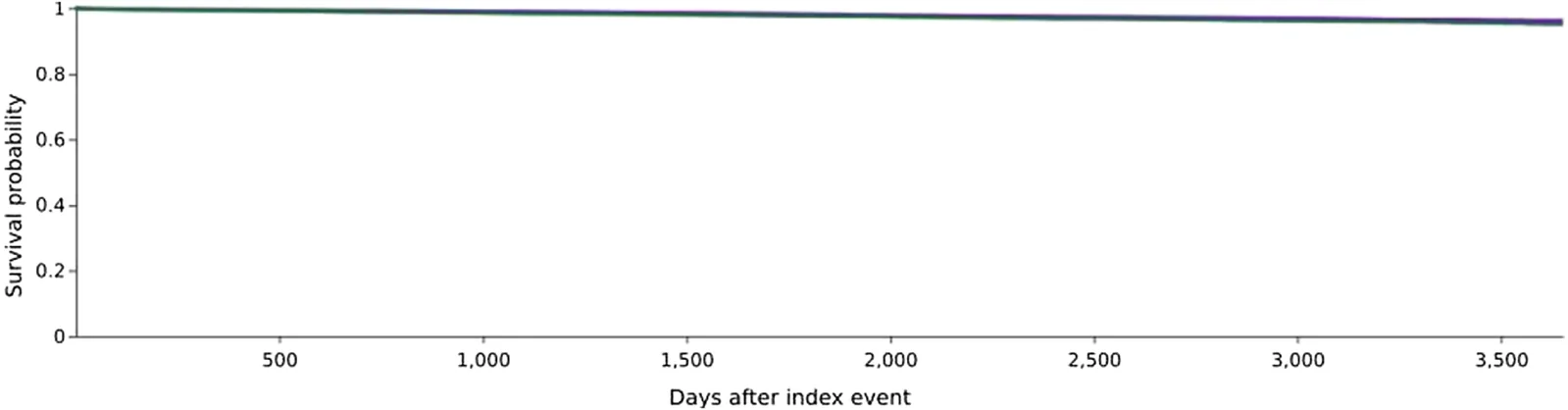
Kaplan–Meier curve for all-cause mortality over 10 years in matched narcolepsy type 1 and type 2 cohorts.

**Table 2:** Comparative risk of mortality and cardiovascular outcomes in matched narcolepsy type 1 and narcolepsy type 2 cohorts.

Outcome	Narcolepsy type 1 (events/at-risk *n*, %)	Narcolepsy type 2 (events/at-risk *n*, %)	Hazard ratio (95% CI)	*P* value
**Primary outcome**				
All-cause mortality	312/19 893 (1.57%)	400/19 892 (2.01%)	0.764 (0.659–0.885)	<0.001
**Secondary outcomes**				
Ischemic stroke	293/19 620 (1.49%)	241/19 610 (1.23%)	1.193 (1.006–1.415)	0.042
Major adverse cardiovascular events	405/19 504 (2.08%)	366/19 488 (1.88%)	1.084 (0.941–1.249)	0.264
Arrhythmia	301/19 584 (1.54%)	339/19 504 (1.74%)	0.864 (0.740–1.009)	0.065
Sudden cardiac death	31/19 895 (0.16%)	36/19 878 (0.18%)	0.844 (0.522–1.365)	0.489

CI, confidence interval. The at-risk denominator excludes patients with the corresponding outcome documented before the start of follow-up.

### Negative-control outcomes

Exploratory analyses of dementia, depression, anxiety, hypothyroidism, and type 1 diabetes mellitus did not show statistically significant differences between cohorts. These findings do not exclude residual bias, but they suggest that the main observed associations were not simply driven by uniformly higher coded-event capture in one phenotype across all outcomes.

## Discussion

In this large propensity score-matched, real-world cohort, NT1 was associated with lower all-cause mortality but a higher risk of ischemic stroke compared with NT2, while no statistically significant differences were observed in MACE, arrhythmia, or sudden cardiac death. These results suggest that narcolepsy phenotypes may not share a uniform long-term risk profile and that phenotype-specific assessment may be clinically relevant.

The lower mortality observed in NT1 was unexpected, given the common assumption that NT1 represents the more biologically severe phenotype. Prior studies examining narcolepsy overall have reported mixed findings regarding mortality, with some describing increased risk of death and others showing attenuation after adjustment^[^9,17–19^]^. One possible explanation is that NT2, as coded in routine practice, captures a more heterogeneous population that may include patients with competing psychiatric or medical burdens associated with poorer overall survival. This interpretation is consistent with reports of greater psychiatric comorbidity in NT2 and in narcolepsy populations more broadly^[^20–22^]^. However, our study cannot determine the specific mechanisms responsible for the mortality difference.

By contrast, NT1 showed a modest but statistically significant increase in the risk of ischemic stroke. This association is biologically plausible because hypocretin deficiency has been linked to altered autonomic regulation, non-dipping nocturnal blood pressure, and other cardiovascular regulatory abnormalities^[^5,12,23–27^]^. Experimental data also suggest that orexin-A may exert neuroprotective effects during cerebral ischemia-reperfusion injury^[^28–30^]^. Taken together, these observations support the possibility that NT1 carries a specific cerebrovascular vulnerability. Nevertheless, the magnitude of the association was small, and the finding should be interpreted cautiously, pending external validation and confirmation in prospective, phenotype-confirmed cohorts.

We did not observe significant differences in broader cardiac endpoints such as MACE, arrhythmia, or sudden cardiac death. This may indicate that shared cardiometabolic risk factors across narcolepsy phenotypes dominate risk for these outcomes, whereas stroke risk may be more sensitive to phenotype-specific neurobiological mechanisms. Prior work has identified elevated cardiovascular burden across narcolepsy populations and supports continued cardiovascular prevention in both phenotypes^[^8,10,11,31–33^]^.

This study has several strengths, including a large, multicenter, real-world data source, long follow-up, and matched comparisons between phenotypes. However, important limitations must be acknowledged. Foremost, NT1 and NT2 were defined solely from ICD-10-CM diagnosis codes (G47.411 and G47.419), without confirmation by polysomnography, multiple sleep latency testing, or cerebrospinal fluid hypocretin-1 measurement, none of which are reliably captured in the federated platform. Phenotype assignment from coded data is therefore susceptible to misclassification, and this concern is greatest for NT2, which is recognized as a diagnostically unstable category that may be reclassified to NT1, idiopathic hypersomnia, or other conditions over time^[^4^]^. Non-differential misclassification of phenotype would generally be expected to bias comparisons toward the null and thus to dilute true between-group differences, implying that the modest ischemic stroke association observed here may be conservative; however, differential misclassification cannot be excluded and could bias estimates in either direction. More rigorous phenotype ascertainment, for example, requiring repeated or persistent diagnosis codes, restricting to patients with documented sleep-specialist encounters, or incorporating medication-supported phenotyping such as cataplexy-directed therapy, would strengthen confidence in these contrasts and should be a priority for confirmatory studies; such analyses could not be implemented within the present de-identified, aggregate-level dataset and were beyond the scope of this study. The analysis also relied on coded EHR data for outcomes and covariates and therefore remains vulnerable to incomplete capture of events and residual confounding. We lacked granular information on cause-specific mortality, medication adherence over time, blood pressure patterns, and other mechanistic variables. Although matching reduced measured confounding, unmeasured confounding cannot be excluded, and causal inference is not possible.

Overall, these findings are best viewed as hypothesis-generating. They support more careful, phenotype-aware risk assessment in narcolepsy and justify future prospective studies with validated phenotyping, longitudinal medication data, and better adjudication of vascular outcomes. More broadly, integrating large-scale, real-world datasets with computational and data-driven methods – including machine-learning and multimodal approaches – may help refine phenotype definitions and improve individualized risk prediction across neurologic and psychiatric disorders^[^34^]^.

## Conclusion

Among adults with narcolepsy in a large, matched, EHR-based cohort, NT1 was associated with lower all-cause mortality but a higher risk of ischemic stroke than NT2 over 10 years. No significant differences were seen in MACE, arrhythmia, or sudden cardiac death. These results should be interpreted cautiously and primarily as hypothesis-generating, while also supporting future prospective work and phenotype-aware clinical risk assessment.

## Data Availability

The data that support the findings of this study are available through the TriNetX Research Network. Access is subject to TriNetX policies and applicable data use agreements; the data are not publicly available.

## Research registration unique identifying number (UIN)

researchregistry11847.

## References

[1] Krahn LE Pankratz VS Oliver L. Hypocretin (orexin) levels in cerebrospinal fluid of patients with narcolepsy: relationship to cataplexy and HLA DQB1*0602 status. Sleep 2002;25:733–36. 10.1093/sleep/25.7.733 12405608

[2] Heier MS Evsiukova T Vilming S. CSF hypocretin-1 levels and clinical profiles in narcolepsy and idiopathic CNS hypersomnia in Norway. Sleep 2007;30:969–73. 10.1093/sleep/30.8.969 PMC1978385 17702265

[3] Scammell TE. Narcolepsy. N Engl J Med. 2015;373:2654–62. 10.1056/NEJMra1500587 26716917

[4] Cavalli RG Tamagni AEM Van Der Meer J. Narcolepsy type 2 is an unstable diagnosis and idiopathic hypersomnia has the potential for remission: long-term and cross-sectional observations from the bern sleep-wake registry. Sleep 2026;49:zsaf242. 10.1093/sleep/zsaf242 PMC13016678 40810502

[5] Barateau L Dauvilliers Y. Cardiovascular burden of narcolepsy: what have we learned and what do we still need to know? Sleep 2023;46:zsad213. 10.1093/sleep/zsad213 37586395

[6] Rani M Kumar R Krishan P. Role of orexins in the central and peripheral regulation of glucose homeostasis: evidences and mechanisms. Neuropeptides 2018;68:1–6. 10.1016/j.npep.2018.02.002 29472002

[7] Ponziani V Gennari M Pizza F. Growing up with type 1 narcolepsy: its anthropometric and endocrine features. J Clin Sleep Med 2016;12:1649–57. 10.5664/jcsm.6352 PMC5155189 27707443

[8] Mohammadi S Moosaie F Saghazadeh A. Metabolic profile in patients with narcolepsy: a systematic review and meta-analysis. Sleep Med 2021;81:268–84. 10.1016/j.sleep.2021.02.040 33740593

[9] Ohayon MM Black J Lai C. Increased mortality in narcolepsy. Sleep 2014;37:439–44. 10.5665/sleep.3470 PMC3920308 24587565

[10] Ben-Joseph RH Saad R Black J. Cardiovascular burden of narcolepsy disease (CV-BOND): a real-world evidence study. Sleep 2023;46:zsad161. 10.1093/sleep/zsad161 PMC10566243 37305967

[11] Riaz M Bhattacharjee R Lo-Ciganic W. Narcolepsy and risk of cardiovascular outcomes beyond stimulant use. Sleep 2025;48:zsaf197. 10.1093/sleep/zsaf197 PMC12710213 40657808

[12] Jennum PJ Plazzi G Silvani A. Cardiovascular disorders in narcolepsy: review of associations and determinants. Sleep Med Rev 2021;58:101440. 10.1016/j.smrv.2021.101440 33582582

[13] Eshera YM Gavrilova L Hughes JW. Sleep is essential for cardiovascular health: an analytic review of the relationship between sleep and cardiovascular mortality. Am J Lifestyle Med. 2024;18:340–50. 10.1177/15598276231211846 PMC11082862 38737888

[14] Yin J Jin X Shan Z. Relationship of sleep duration with all-cause mortality and cardiovascular events: a systematic review and dose-response meta-analysis of prospective cohort studies. J Am Heart Assoc. 2017;6:e005947. 10.1161/JAHA.117.005947 PMC5634263 28889101

[15] Palchuk MB London JW Perez-Rey D. A global federated real-world data and analytics platform for research. JAMIA Open. 2023;6:ooad035. 10.1093/jamiaopen/ooad035 PMC10182857 37193038

[16] Agha RA Mathew G Rashid R. Revised strengthening the reporting of cohort, cross-sectional and case-control studies in surgery (STROCSS) guideline: an update for the age of artificial intelligence. Prem J Sci 2025;10:100081.

[17] Cohen A Mandrekar J St Louis EK. Comorbidities in a community sample of narcolepsy. Sleep Med 2018;43:14–18. 10.1016/j.sleep.2017.11.1125 PMC5931205 29482805

[18] Jennum P Thorstensen EW Pickering L. Morbidity and mortality of middle-aged and elderly narcoleptics. Sleep Med 2017;36:23–28. 10.1016/j.sleep.2017.03.029 28735916

[19] Hsu C Yang Y Chen YB. All-cause and cause-specific mortality among patients with narcolepsy. JAMA Netw Open 2025;8:e2536771. 10.1001/jamanetworkopen.2025.36771 PMC12511998 41066120

[20] Østerby NCH Baandrup L Jennum PJ. Psychiatric comorbidity in Danish patients with narcolepsy type 1, narcolepsy type 2, and idiopathic hypersomnia: a case-control study. Sleep Adv 2024;5:zpae073. 10.1093/sleepadvances/zpae073 PMC11489886 39430232

[21] Alasim H AlQazlan S Albanyan S. Comorbid psychiatric disorders among patients with narcolepsy. Sleep Breath 2020;24:629–36. 10.1007/s11325-019-01890-8 31280401

[22] Black J Reaven N Funk S. Medical comorbidity in narcolepsy: findings from the burden of narcolepsy disease study. Sleep Med 2017;33:13–18. 10.1016/j.sleep.2016.04.004 28449892

[23] Kaufmann CN Riaz M Park H. Narcolepsy is associated with subclinical cardiovascular disease as early as childhood: a big data analysis. J Am Heart Assoc 2025;14:e038426. 10.1161/JAHA.124.039899 PMC12229201 40207482

[24] Dauvilliers Y Jaussent I Krams B. Non-dipping blood pressure profile in narcolepsy with cataplexy. PLoS One 2012;7:e38977. 10.1371/journal.pone.0038977 PMC3387234 22768053

[25] Wang J Yan Z Dong X. Diurnal changes in blood pressure and heart rate in children with narcolepsy with cataplexy. J Sleep Res 2023;32:e13736. 10.1111/jsr.13736 36163423

[26] Fronczek R Overeem S Reijntjes R. Increased heart rate variability but normal resting metabolic rate in hypocretin-orexin-deficient human narcolepsy. J Clin Sleep Med 2008;4:248–54. PMC2546458 18595438

[27] Hublin C Matikainen E Partinen M. Autonomic nervous system function in narcolepsy. J Sleep Res. 1994;3:131–37. 10.1111/j.1365-2869.1994.tb00119.x 10607117

[28] Xu D Kong T Cheng B. Orexin-A alleviates cerebral ischemia-reperfusion injury by inhibiting endoplasmic reticulum stress-mediated apoptosis. Mol Med Rep 2021;23:1. 10.3892/mmr.2021.11905 PMC7893697 33576468

[29] Xu D Kong T Shao Z. Orexin-A alleviates astrocytic apoptosis and inflammation via inhibiting OX1R-mediated NF-κB and MAPK signaling pathways in cerebral ischemia-reperfusion injury. Res Sq 2021;1867:166230. 10.1016/j.bbadis.2021.166230 34358627

[30] Zhu M Li X Guo J. Orexin A protects against cerebral ischemia-reperfusion injury by enhancing reperfusion in ischemic cortex via HIF-1α-ET-1/eNOS pathway. Brain Res Bull 2024;218:111105. 10.1016/j.brainresbull.2024.111105 39442584

[31] Tao Y Luo J Xu Y. Narcolepsy and cardiovascular disease: a two-sample Mendelian randomization study. Sleep Med 2024;113:6–12. 10.1016/j.sleep.2023.10.029 37976908

[32] Futenma K Takaesu Y Nakamura M. Metabolic syndrome-related comorbidities in narcolepsy spectrum disorders: a preliminary cross-sectional study in Japan. Int J Environ Res Public Health 2022;19:6285. 10.3390/ijerph19106285 PMC9141676 35627822

[33] Kwon Y Gami AS Javaheri S. Cardiovascular risks in people with narcolepsy: expert panel consensus recommendations. J Am Heart Assoc 2024;13:e035168. 10.1161/JAHA.124.035168 PMC11963943 39119988

[34] Narimani M Naeim M. Artificial intelligence in mental health: integrating opportunities and challenges of multimodal deep learning for mental disorder prevention and treatment. Ann Med Surg 2025;87:5757–61. 10.1097/MS9.0000000000003624 PMC12401332 40901142

